# Development of Lily Starch Films Reinforced with Chitosan–Honeysuckle Essential Oil Hybrid Particles and Cellulose Nanofibers for Enhanced Properties

**DOI:** 10.3390/foods14040589

**Published:** 2025-02-10

**Authors:** Yuchen Liu, Haishan Xu, Ziyi Chen, Ziyi Xie, Hui Wen, Xia Chang, Gaoyang Li

**Affiliations:** 1 Longping Branch, College of Biology, Hunan University, Changsha 410125, China; lyc000627@163.com (Y.L.); 17708459423@163.com (H.X.); ziyichen@hnu.edu.cn (Z.C.); xiezy566@163.com (Z.X.); wenhuiwinnie@163.com (H.W.); 2 Hunan Provincial Key Laboratory for Fruits and Vegetables Storage Processing and Quality Safety, Hunan Agricultural Product Processing Institute, Hunan Academy of Agricultural Sciences, Changsha 410125, China; 3 College of Biology, Hunan University, Changsha 410082, China

**Keywords:** lily starch films, hybrid composite, cellulose nanofibers, biodegradable packaging, green materials

## Abstract

To address the limitations of current starch-based food packaging materials, this study develops a novel sustainable material—honeysuckle hybrid particle-enhanced starch active fiber film (LNC). Derived from lily starch, this film is a promising green material for food preservation. The film’s functionality was enhanced by integrating honeysuckle essential oil and chitosan–ZnO composite hybrid particles, while cellulose nanofibers were used to create a stable network structure. Honeysuckle essential oil was analyzed, identifying 40 main compounds, with linalool as the predominant component (48.41%). Subsequently, honeysuckle essential oil hybrid particles (CZH) were successfully developed. Using lily starch as the matrix, the effects of honeysuckle essential oil, CZH, and cellulose nanofibers (CNF) on the film’s properties were investigated, leading to the fabrication of functional composite films (LNCs). The results indicated that CZH and CNF significantly enhanced the molecular structure, crystallinity, thermal stability, surface hydrophobicity (contact angle θ > 103°), and tensile strength (37.31 MPa) of the films. Additionally, CZH improved the film’s UV-blocking capacity (UV-blocking rate of 85.92%), and LNC exhibited superior gas barrier properties. This study demonstrates that lily starch-based composite films possess exceptional mechanical, optical, and barrier properties, thereby highlighting their potential for use in functional food packaging applications.

## 1. Introduction

Food packaging plays a key role by forming a protective layer around the food. It helps keep the quality and safety, and extends the product’s shelf life, preventing damage during transportation and storage from chemical, biological, environmental, or physical factors [1,2]. However, most food packaging uses flexible petroleum-based plastics, which have low degradability and biodegradability, causing serious environmental threats and harming biodiversity. The consumption of synthetic polymers increased over time. By 2050, non-biodegradable plastic waste is projected to exceed 25 billion tons [3], and the plastics sector could contribute to 15% of global carbon emissions [4]. The accumulation of synthetic polymer waste leads to plastic debris in the environment, posing serious threats to both nature and human health.

Bio-based films composed of natural polysaccharides, proteins, lipids, or their blends are emerging biodegradable materials designed to address existing challenges. Compared to proteins and lipids, polysaccharide-based films have higher stability, abundant sources, and lower costs [5]. These films function as delivery systems for natural bioactive compounds, including antioxidants and antimicrobials, enabling controlled release. Starch, cellulose, and chitosan are widely available natural polysaccharides with excellent biocompatibility, eco-friendliness, non-toxicity, and film-forming properties, making them highly promising for developing biodegradable film materials [6].

Although a range of biodegradable food packaging materials has been developed, such as polylactic acid (PLA) and konjac glucomannan (KGM), these substances often encounter difficulties regarding mechanical strength, flexibility, and environmental effects during manufacturing and disposal. For instance, polylactic acid (PLA) is relatively brittle and requires an energy-intensive synthesis procedure, which limits its sustainability [7]. Conversely, although KGM possesses some flexibility, it is costly and lacks the necessary strength for certain packaging uses [8]. In contrast, films based on the natural polysaccharide lily starch offer a promising alternative. Among the common polysaccharides, lily starch (LS) shows great potential in making films because of its special properties. It is a small-granule starch with a large surface area and high water affinity [9]. These features improve the film’s strength and flexibility. Its low gelatinization temperature and good processability made it suitable for producing biodegradable films [10]. These films could be useful for food packaging and have potential in functional materials. However, there is little research on using lily starch as a base for green food packaging films. Chitosan (CS), derived from chitin, is a widely available, biodegradable, and non-toxic natural polymer with antimicrobial properties [11]. It is widely used in biomedical fields and, in recent years, also in active food packaging. However, due to its poor flexibility and mechanical strength, it is often combined with other polymers, such as starch and cellulose, to create high-quality composite films [12]. In addition, cellulose nanofibers (CNF) are an emerging green natural material with excellent mechanical properties, film-forming ability, and low permeability [13]. Their adjustable surface chemistry makes them an ideal material for preservation films, greatly enhancing barrier performance and prolonging food shelf life [14].

Natural plant essential oils are widely used in active food packaging films. Common essential oils, such as clove, thyme, oregano, ginger, and citrus peel oils [15,16], serve as active components to provide antibacterial and antioxidant functions. Honeysuckle, a traditional herb, has anti-inflammatory, antioxidant, antipyretic, hepatoprotective, and choleretic effects [16]. Its essential oil has been proven to have antibacterial, antioxidant, and larvicidal activities but has not been applied in active packaging [17]. Moreover, due to the volatility and instability of essential oils, it is challenging to provide functional properties to packaging films under conventional conditions, requiring new techniques for protection and controlled release. The preparation of polysaccharide–metal–essential oil hybrid particles has proven to be an effective strategy [18]. Among these, hybrid particles formed by chitosan, cyclodextrin, and cellulose combined with ZnO were extensively studied for their low synthesis cost and strong antibacterial properties [19].

The performance of starch-based films can typically be enhanced through crosslinking techniques that strengthen intermolecular interactions [20]. By combining honeysuckle essential oil with chitosan to create hybrid particles, these particles may improve the film’s antibacterial and antioxidant properties [21]. Additionally, the combination of lily starch and cellulose nanofibers could potentially increase the film’s mechanical durability, flexibility, and barrier effectiveness [22]. Through these crosslinking interactions, we anticipate the film will demonstrate better mechanical properties, barrier efficiency, antibacterial performance, and water resistance, thus fulfilling the dual requirements of functionality and environmental sustainability in contemporary food packaging [23].

This study first identified the main chemical components of honeysuckle essential oil using GC-MS. Then, CS-ZnO hybrid particles loaded with the essential oil were prepared and their physicochemical properties were characterized. These hybrid particles were added to a natural food packaging film based on lily starch, and cellulose nanofibers were introduced to enhance film properties. A novel bioactive packaging film was developed and its physicochemical properties, optical performance, and thermal stability were evaluated. This study provides new ideas for developing and applying active food packaging materials, expanding material sources, and promoting functional and eco-friendly packaging. Moreover, by integrating the synergistic effects of natural polysaccharides, essential oils, and nanofibers, this study not only enhances the performance of packaging films but also offers an innovative approach to tackling sustainability and environmental challenges in the food packaging industry.

## 2. Materials and Methods

### 2.1. Materials

Lily starch was extracted from lily bulbs using a method adapted from Zhang et al. [24], with minor modifications. The specific steps are as follows: The lily bulbs were crushed using a high-speed grinder and the mixture was then filtered using a 100-mesh nylon cloth to obtain the filtrate. The filtrate was centrifuged at 5000× *g* for 10 min at 25 °C, and the supernatant was discarded, retaining the starch slurry at the bottom. The precipitated starch slurry was washed with NaOH (0.05 M), stirring every 30 min. The NaOH solution was replaced every 3 h until the washing solution turned colorless. Finally, the washed starch was freeze-dried and passed through an 80-mesh sieve.

Honeysuckle essential oil was purchased from Ji An Huatianbao Herbs Biological Products Factory (Ji’an, China). Chitosan was purchased from Shanghai Macklin Biochemical Co., Ltd. (Shanghai, China). Carboxylated cellulose nanofibers were procured from Qihong Technology Co., Ltd. (Guangxi, China). Zinc acetate, sodium hydroxide, sodium tripolyphosphate, glycerol, and other chemicals were obtained from Sinopharm Chemical Reagent Co., Ltd. (Shanghai, China). All reagents used were of analytical grade, and the water used was either Milli-Q or deionized.

### 2.2. GC-MS Analysis of the Honeysuckle Essential Oil

The study used a QP2010 Ultra GC-MS instrument (Shimadzu, Kyoto, Japan) to analyze honeysuckle essential oil samples diluted 50 times with methanol. The analysis utilized a DB-5MS column (Agilent, J&W Scientific, Santa Clara, USA, 30 m × 0.25 mm × 0.25 μm), with the injector temperature maintained at 280 °C and a split ratio of 10:1. Helium (high purity) served as the carrier gas at a 2.50 mL/min flow rate. The column temperature program commenced at 40 °C for 2 min, then increased by 10 °C/min to 280 °C. The ion source temperature was 220 °C, while the interface was set to 280 °C. A solvent cut time of 3 min was applied, and mass spectra were recorded within an *m*/*z* range of 33–500, with spectral matching performed using the NSIT library database.

### 2.3. Preparation of CS–ZnO@HEO (CZH) Hybrid Particles 

The preparation of CS–ZnO@HEO hybrid particles was slightly modified based on the previously reported sol–gel technique by Tilak Gasti, et al. [21]. Specifically, 0.5 g CS was dissolved in a 2% (*V*/*V*) acetic acid solution and stirred at 4000 rpm using a high-speed homogenizer (SCIENTZ-IID, Ningbo Scientz Biotechnology Co., Ltd., Ningbo, China). The solution was filtered to remove insoluble substances. Then, 30 mL of 0.68 M zinc acetate solution was added and mixed for 15 min. Next, 2 mL of honeysuckle essential oil and 0.2 g of sodium tripolyphosphate (TPP) were incorporated. The pH of the solution was adjusted to 11 using a suitable base at 70 °C using 11.25 M NaOH. The resulting yellow precipitate was filtered, redispersed, and dispersed at 10,000 rpm for 5 min. The precipitate was then centrifuged at 6000 rpm for 30 min and washed with ultrapure water until the pH reached neutral. Finally, it was dried at 45 °C for 4–5 h and stored in the dark. The same process was used to prepare CS and CS-ZnO as controls. 

### 2.4. Characterization of CS–ZnO@HEO (CZH) Hybrid Particles 

#### 2.4.1. Fourier-Transform Infrared Spectroscopy (FTIR) Analysis

The hybrid particles (CS, CS-ZnO, CZH) (1.5 mg) were evenly mixed with KBr (198.5 mg) and pressed into tablet form. The spectra were recorded in the range of 4000–400 cm⁻^1^ using a Fourier transform infrared spectrometer (Thermo Fisher Scientific Nicolet iS20, Waltham, MA, USA).

#### 2.4.2. X-Ray Diffraction (XRD) Analysis

The XRD pattern of hybrid particles was collected by a D8 Advance X-ray diffractometer (XRD, Rigaku, Tokyo, Japan) equipped with Cu Kα radiation generated at 40 kV and 30 mA. The scanning scope of 2θ ranged from 5 to 80° at a rate of 5°/min.

#### 2.4.3. Scanning Electron Microscopy (SEM) Analysis

The microstructures of the samples were analyzed using a scanning electron microscope (SEM). Freeze-dried gel samples (2 mg) were gold sputter-coated and affixed to an aluminum mount for imaging at an operating voltage of 3.0 kV.

### 2.5. Preparation of Lily Starch-Based Films with Different Compositions

**Figure 1:**
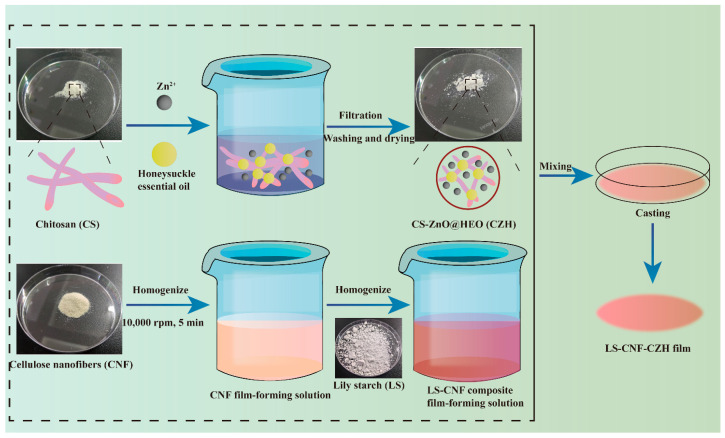
Simplified preparation flowchart of CZH hybrid particles and LNC films.

Lily starch-based films with different compositions were prepared using the solvent casting method. A simplified preparation flowchart is shown in Figure 1. In short, 0.25 g of cellulose nanofibers (CNF) was added to 49 g of water at room temperature and homogenized at 10,000 rpm for 5 min to fully disperse it. Then, 0.05 g of hybrid particles (CZH) was added and homogenized again at 10,000 rpm, for 5 min. Next, 1 g of lily starch and 0.3 g of glycerol were added, followed by another 5 min of homogenization to obtain the film solution. The solution was transferred into disposable plastic Petri dishes and dried at 45 °C for 6 h to form the LNC film. The ambient relative humidity was standardized to 50% prior to drying. Other films with different compositions were prepared using the same method: LS was a pure lily starch film, LSE was a lily starch film with honeysuckle essential oil, LSC was a film without CNF, and LSN was a film without CZH hybrid particles.

### 2.6. Characterization of Lily Starch-Based Films with Different Compositions

#### 2.6.1. FTIR and XRD Analysis

The analysis methods for FTIR and XRD were generally the same as described in Section 2.4.1 and Section 2.4.2.

#### 2.6.2. Thermal Stability Analysis (DSC and TGA)

The thermal stability (DSC and TGA) of lily starch-based films with varying compositions was evaluated using a thermogravimetric analyzer (NETZSCH5, TA Instruments, New Castle, DE, USA). Approximately 4 mg of the film sample was placed in a ceramic crucible and heated from 35 °C to 800 °C at a constant rate of 10 °C per minute under a nitrogen atmosphere.

#### 2.6.3. Film Thickness

The thickness of the films was determined using a digital micrometer (Mitutoyo Absolute, Tester Sangyo Co., Ltd., Nagahama, Japan) with a precision of 0.001 mm. Each film sample was cut into five strips (10 mm × 50 mm), and the thickness was measured at three positions on each strip (both ends and the center). The average thickness was calculated, and uniformity was evaluated accordingly. 

#### 2.6.4. Optical Properties Analysis

Using air as the reference, the UV–Vis transmittance of the different components films was recorded over a wavelength range of 200–800 nm using a UV–Vis spectrophotometer (UV-1800 UV–Visible Spectrophotometer, Shimadzu Instruments, Suzhou, China). The opacity of each film was evaluated at 600 nm absorbance using the following formula [25]:

$$\mathrm{Opacity}\;\left({\mathrm{mm}}^{-1}\right)=\frac{\mathrm{Absorbance}}{t}$$

where the *t* was the film thickness (mm).

The color of the films was measured using an XE-type fully automated colorimeter. A white standard color plate (L* = 91.85, a* = −0.78, b* = 2.78) was used as the background. The L (lightness), a (red-green), and b (yellow-blue) values were recorded, and the total color difference (ΔE) of the sample was calculated using the following formula:

$$∆E=\sqrt{{\left(L^{*}-L\right)}^{2}{+(a^{*}-a)}^{2}{+(b^{*}-b)}^{2}}$$

#### 2.6.5. Scanning Electron Microscopy (SEM) Analysis

The same method as used in Section 2.4.3 above.

#### 2.6.6. Tensile Strength (TS) and Elongation at Break (EB) Analysis

The films were cut into strips of 10 mm × 50 mm, and the tensile strength (TS) and elongation at break (EB) were tested using the texture analyzer CT3 (Brookfield Engineering Labs, Middleborough, MA, USA) with roller clamps TA-RCA, fixed with an initial distance of 30 mm. The test was conducted at a stretching rate of 50 mm/min, and the TS and EB of the films were calculated using the following formulas:

$$\mathrm{TS}\;\left(\mathrm{MPa}\right)=\frac{F_{\max}}{s}$$

$$\mathrm{EB}\;\left(\%\right)=\frac{∆L}{L_{0}}\times 100$$

where *F_max_* represents the maximum force at film fracture (N), *S* is the cross-sectional area of the film (mm^2^), and Δ*L* and *L*_0_ refer to the elongation of the film and the initial clamp length (mm), respectively.

#### 2.6.7. Water Contact Angle Analysis

In summary, at room temperature, a contact angle analyzer was used to drop 10 μL of distilled water onto the film surface. The apparent shape of the water droplet on the composite film was photographed and recorded, and the water contact angle (WCA) was calculated to evaluate the hydrophobicity of the film surface. Each sample was tested in triplicate.

#### 2.6.8. Determination of Gas Permeability Properties and Water Vapor Permeability

The water vapor permeability (WVP) of the film was measured following the method outlined by Zhang et al. [26]. First, anhydrous calcium chloride was placed at the bottom of a centrifuge tube, and the tube was sealed with the film. The initial weight of the centrifuge tube was recorded, and the tubes were placed in a constant temperature and humidity chamber (relative humidity: 75%, temperature: 25 °C) for 48 h. The WVP of the film was calculated using the following formula:

$$\mathrm{WVP}\;\left(\mathrm{kg}\cdot m^{-1}\cdot s^{-1}\cdot {\mathrm{Pa}}^{-1}\right)=\frac{∆w\times L}{∆t\times A\times ∆p}$$

The parameters were defined as follows: Δ*w* (kg) represented the mass reduction via film permeation, Δ*t* (s) denoted the time required to reach equilibrium, and *L* (m) corresponded to the film’s thickness. The parameter *A* (m^2^) was specified as the film’s effective coverage area (0.00049 m^2^), while Δ*p* (Pa) indicated the differential water vapor pressure across the film during experimental conditions.

Oxygen permeability (OP) and carbon dioxide permeability (CDP) were determined using the protocol established by Chang et al. [27]. A centrifuge tube (2.8 cm diameter × 11.5 cm height) containing 5 g of deoxidizer and potassium hydroxide was sealed with the composite film. Following initial mass measurement, all assemblies were transferred to a climate-controlled chamber (25 °C, 75% RH) for 48 h of equilibration. The OP and CDP of the composite film were calculated as follows:

$$\mathrm{OP}\;\left(\mathrm{kg}\cdot m^{-1}\cdot s^{-1}\cdot {\mathrm{Pa}}^{-1}\right)=\frac{∆w\times L}{∆t\times A}$$

$$\mathrm{CDP}\;\left(\mathrm{kg}\cdot m^{-1}\cdot s^{-1}\cdot {\mathrm{Pa}}^{-1}\right)=\frac{∆w\times L}{∆t\times A}$$

where Δ*w* (kg) represents the weight loss through the film, Δ*t* (s) denotes the equilibrium time, *L* (m) is the thickness of the film, and *A* (m^2^) is the effective area covered by the film (0.00049 m^2^). All samples were tested in triplicate for accuracy.

### 2.7. Statistical Analysis

The statistical data were determined by SPSS 27.0 (SPSS Inc., Chicago, IL, USA). The statistical data were evaluated by one-way analysis of variance (ANOVA) and the Duncan test. Statistical difference was defined as *p* < 0.05.

## 3. Results and Discussion

### 3.1. Identification of Compounds in Honeysuckle Essential Oil

**Figure 2:**
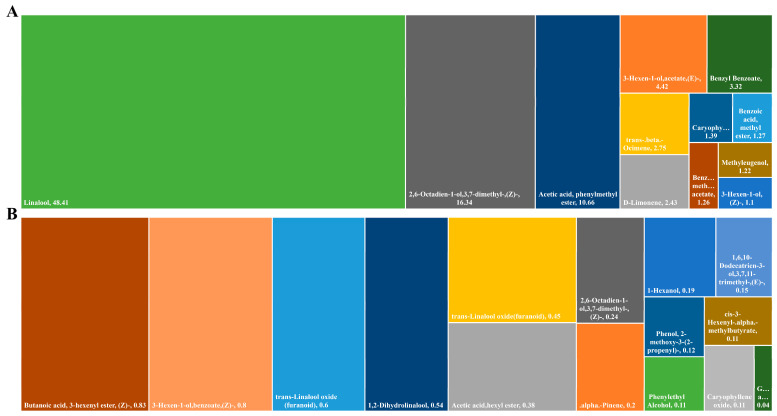
The tree diagram of the main components of honeysuckle essential oil (**A**: content > 1%, **B**: 0.1% < content < 1%).

**Figure 3:**
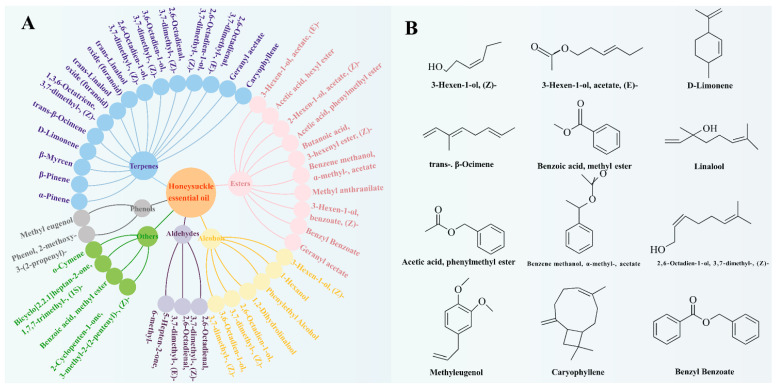
Composition Classification (**A**) and Structural Formulas of Major Components (Content > 1%) of Honeysuckle Essential Oil (**B**).

Figure 2 and Figure 3 show the main compounds identified in honeysuckle essential oil by GC-MS. There were 40 compounds identified in the essential oil, which were roughly divided into six categories: terpenes (15), esters (10), alcohols (6), aldehydes (3), phenols (2), and other compounds (4). The essential oil of honeysuckle mainly contained terpenes, with linalool (48.41%) as the main compound. Other major terpenes include α-pinene, β-pinene, and D-limonene, which give the oil its unique fragrance. This was similar to previous research findings [17]. Esters were the second largest group in the essential oil, mainly including (E)-3-hexen-1-yl acetate and hexyl acetate, which typically have a fresh fruity and floral scent. Alcohols, such as 1-hexanol and phenylethyl alcohol, also make up a certain proportion and give the oil a mild, sweet floral note. Although aldehydes were presented in lower amounts, they contributed significantly to enhancing the overall scent of the oil. (Z)-2,6-octadienal, with its green and citrus aroma, added a fresh touch. Phenolic compounds, such as methyl eugenol and 2-methoxy-3-(2-propene)-phenol, provided spicy and warm scents, making the fragrance more complex [28]. Finally, other compounds, such as benzyl benzoate and caryophyllene, not only improved the stability of the oil but also enriched its aroma. These components make honeysuckle essential oil a complex mixture of natural compounds, which may have various biological activities, such as antioxidant, antibacterial, and antidepressant effects. It has potential applications in fragrances, food flavoring, and functional food packaging.

### 3.2. Brief Characterization of CS-ZnO@HEO (CZH) Hybrid Particles

**Figure 4:**
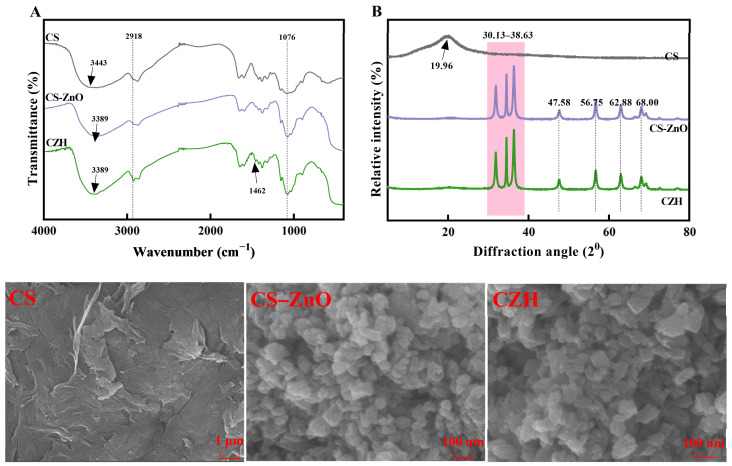
FTIR spectra (**A**) and XRD patterns (**B**), and SEM images of the particles.

The FTIR spectra of the particles are presented in Figure 4A. CS, CS-ZnO, and CZH exhibited significant differences in their spectra. CS exhibited a broad, flat peak at 3443 cm⁻^1^, which corresponds to the O-H and N-H stretching vibrations in the chitosan molecule [29]. After ZnO hybridization, this peak shifted to 3389 cm⁻^1^, and in CZH, this peak became narrower and deeper, which might be related to the hydroxyl groups in alcohols present in honeysuckle essential oil [30]. Moreover, new peaks appeared at 2918 cm⁻^1^ and 1462 cm⁻^1^ in CZH, which can be ascribed to the presence of terpenes in the essential oil, especially the methylene and methyl C-H stretching vibrations of linalool [31], the major component. These results indicated that the ZnO hybrid particles were successfully formed, and the honeysuckle essential oil was successfully incorporated into the CZH hybrid particles.

The XRD patterns of the particles are shown in Figure 4B. Pure chitosan (CS) exhibited a characteristic diffraction peak at a (2θ) angle of 19.96°, corresponding to the reflection of the (110) crystallographic plane of chitosan. CS-ZnO and CZH displayed three prominent crystalline peaks in the range of 30.13–38.63°, which correspond to the reflections of the (100), (002), and (101) planes of zinc oxide. These characteristic peaks represent the zincite phase of ZnO particles. Additionally, diffraction peaks were observed at 47.58°, 56.75°, 62.88°, and 68°, corresponding to the (102), (110), (103), and (112) planes, respectively. After the incorporation of essential oil, no significant changes were observed in the XRD patterns of CZH, which is consistent with previous studies [21].

The SEM images of the prepared CS, CS-ZnO, and CZH hybrid particles are shown in Figure 4. The CS particles exhibited relatively smooth surfaces, attributed to the dense polysaccharide chains. In contrast, the CS-ZnO and CZH particles displayed asymmetrical crystalline 3D morphologies, with the bright white regions on the crystalline particles representing CS molecules adhered to Zn^2+^ [21]. Moreover, compared to CS-ZnO, the CZH particles did not show significant structural changes, indicating that the addition of the essential oil did not substantially disrupt the structure of the hybrid particles. In summary, this study successfully prepared hybrid particles CZH encapsulating honeysuckle essential oil.

### 3.3. FTIR and XRD Analysis of Films

**Figure 5:**
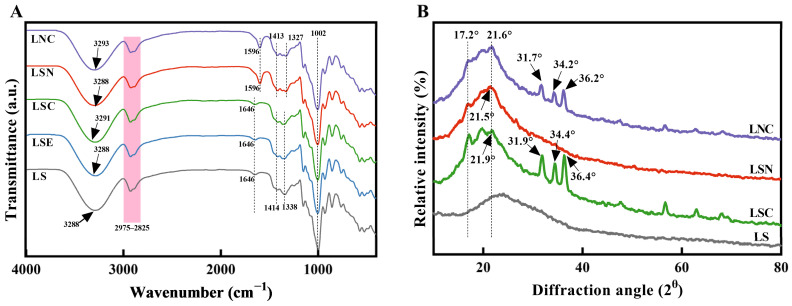
FTIR spectra (**A**) and XRD patterns (**B**) of different films.

Figure 5A shows the FTIR spectra of lily starch films with different components, revealing potential interactions between LS, CNF, and CZH (chitosan–ZnO@honeysuckle essential oil hybrid particles). LS, LSE, and LSN all showed a characteristic peak at 3288 cm⁻^1^, which was clearly attributable to O-H stretching vibrations [32]. This indicated that the direct addition of honeysuckle essential oil and CNF did not significantly disturb the O-H bonds in lily starch, and there was little or no hydrogen bonding. However, after adding the hybrid particles CZH to the LSC and LNC films, the O-H stretching peaks shifted to 3291 cm⁻^1^ and 3293 cm⁻^1^, respectively. It was speculated that the CZH hybrid particles formed hydrogen bonds with lily starch and CNF [13], which helped retain and slowly release the essential oil in the films. All films exhibited characteristic absorption bands within the 2975–2825 cm⁻^1^ range, attributed to symmetrical and asymmetrical C-H stretching vibrations in methylene groups [13]. LS, LSE, and LSC showed a characteristic peak at 1646 cm⁻^1^, while after the addition of CNF, this peak shifted to 1596 cm⁻^1^, corresponding to the C-O stretching (amide-I) group [33]. A similar result was observed in the characteristic peak representing the C-H stretching vibration of methyl (-CH₃) groups [21]. LS, LSE, and LSC showed this peak at 1338 cm⁻^1^, while after the addition of CNF, the peak shifted to 1327 cm⁻^1^. It is speculated that the addition of CNF might have led to changes in the molecular arrangement or structural reorganization, resulting in a shift in the peak.

X-ray diffraction analysis was used to determine the crystallinity or amorphous properties of the films with different components. Their XRD patterns are shown in Figure 5B. The pure LS film showed a broad peak at 23.7°, which corresponded to the amorphous structure of the starch matrix, similar to the typical diffraction pattern of natural starch [34]. When CNF and CZH particles were added to the LSC, LSN, and LNC films, the XRD patterns changed significantly. Specifically, the characteristic peak of LS in the LSC film shifted to 21.9°. This indicates that the CZH particles affected the amorphous structure of the starch. Similarly, when CNF were added to the LS film, the diffraction peak shifted to 21.5°. This suggests that the molecular interaction between starch and CNF strengthened. It may have caused a slight structural rearrangement [35]. The appearance of new diffraction peaks at 31.7°, 34.2°, and 36.4° in the LSC (likely a composite material, e.g., La-Sr-Co oxide) and LNC (another composite, e.g., La-Ni-Co oxide) films corresponds to the (100), (002), and (101) crystal planes of zinc oxide (ZnO) within CZH particles [21]. These peaks showed that crystalline ZnO was successfully incorporated into the starch matrix, which increased the overall crystallinity of the composite films. Overall, the addition of CNF and CZH particles significantly affected the molecular arrangement and crystallinity of the LS films. Among them, the LNC film showed the most significant structural change due to the contribution of crystalline ZnO in the CZH particles. This structural enhancement could help improve the mechanical properties and functionality of the films.

### 3.4. Thermal Stability Analysis of Films

**Figure 6:**
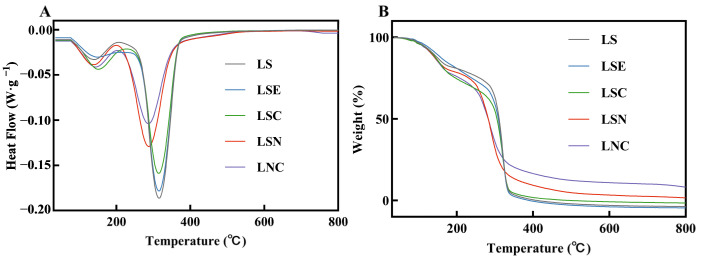
Thermal stability analysis of different films: DSC (**A**), TGA (**B**).

The DSC and TGA thermograms of films with varying compositions are displayed in Figure 6. The initial weight loss of the films occurred between 52.67 °C and 72.35 °C, which was attributed to the evaporation of water [36]. In the temperature range of 105.29–187.85 °C, all films showed a second significant weight loss, and the films with added CZH and CNF showed more noticeable heat release in this range. This was similar to previous studies, which suggested that it was due to the continued loss of water and the structural rearrangement or glass transition of cellulose nanofibers and chitosan during heating [37]. The third stage of weight loss for the films occurred between 200.68 °C and 342.28 °C, during which the most significant weight loss was observed. In the DSC curves, the temperature at which the maximum heat release occurred varied considerably across the samples. The heat release peaks for LNC and LSN appeared at 290.09 °C, while the peaks for LS, LSE, and LSC were delayed to 318.75 °C. This could be due to the interaction between CNF and other components, which affected the thermal stability of the composite materials [37]. Specifically, CNF might have formed hydrogen bonds or physical cross-links with chitosan or other components, which could lower the thermal degradation temperature of the materials [38]. Some studies have shown that the introduction of CNF can cause changes in the crystalline regions of the composite materials, thus affecting their thermal degradation behavior [39]. For example, CNF might make the structure of the composite material more compact, lowering the onset temperature of thermal decomposition.

### 3.5. Optical Properties—Film Thickness, Light Transmittance, and Color

**Figure 7:**
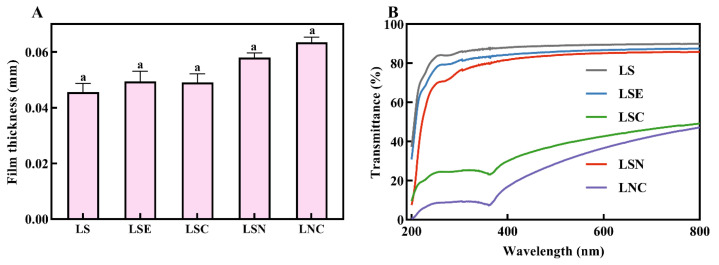
The thickness of different films (**A**) and light transmission characteristics (**B**). Lowercase letters in the same column indicate significant differences (*p* < 0.05).

The thickness of the films with different compositions is shown in Figure 7A. The pure LS film has the smallest thickness, and as other substances are added, the film thickness gradually increases. The LNC film, which contained both CNF and CZH, had the largest thickness. However, according to the significance analysis, no substantial difference was found in the thickness of these five films. As a result, the inclusion of CNF and CZH had a negligible effect on the film thickness, which aligns with results reported in earlier studies [40].

The optical properties of food packaging films play a crucial role in influencing consumer preference [41], and food is prone to deterioration when exposed to ultraviolet light. Therefore, the UV resistance of food packaging films is also an important consideration [42]. The UV–visible transmittance of film samples with different compositions, collected in the range of 200–800 nm, is shown in Figure 7B. The pure LS film had the worst UV barrier performance. In the UV range of 200–400 nm, the UV transmittance of LS film exceeded 92%. Adding honeysuckle essential oil and CNF to the LS film hardly improved its UV resistance. In contrast, the LS films with CZH hybrid particles (i.e., LSC and LNC) showed excellent UV blocking ability. At 368.85 nm, the UV transmittance of the LSC film was only 38.08%, while the LNC film had a UV transmittance of 15.08% at the same wavelength. These results proved that CZH had UV-blocking ability, and the interaction between CNF, CZH, and LS made the film more compact, reducing the transmittance [40].

The color of the films with varying compositions was further analyzed using color parameters. The results are shown in Table 1. In terms of lightness, LNC and LSC achieved the best L values, 40.88 ± 2.24 and 40.11 ± 1.73, respectively, indicating that these two films became clearer and more transparent after packaging. At the same time, these two films also displayed lower yellowness, with b values of −5.98 ± 0.72 and −6.04 ± 0.85, indicating that the colors of these two films are more biased towards blue tones, making them more suitable for packaging cold-toned foods [43].

### 3.6. SEM Analysis of Films

**Figure 8:**
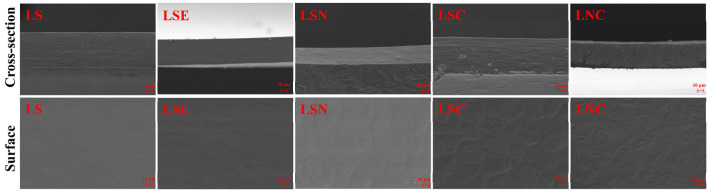
The SEM images of the surface and cross-section of different films.

Figure 8 presents the surface and cross-sectional SEM images of the various films. Distinct morphological variations were observed among the films with different compositions. The pure LS film had an obviously smooth and dense surface, and its cross-section showed a uniform layered structure, indicating a well-organized internal structure with no porosity. In contrast, the surface of the LSE and LSN films was slightly rougher, and their cross-sections displayed a slightly increased thickness with a denser internal structure. This indicates that the direct incorporation of honeysuckle essential oil and CNF improved the structural integrity of the films. The cellulose nanofiber, with its large surface area and numerous hydroxyl groups, could form a complex crosslinked network with the matrix molecules through hydrogen bonding or van der Waals forces, thereby enhancing the tightness and stability of the films [22].

For the LSC and LNC films containing CZH hybrid particles, the surface roughness significantly increased, and their cross-sections showed uneven layered structures with small pores. This may have been due to the uneven dispersion of the CZH particles within the matrix. Additionally, the surface and cross-sectional roughness of the LNC film were higher, indicating that the combined action of CNF and CZH enhanced the internal crosslinking structure of the film. At the same time, the CZH hybrid particles might have contributed to the enhancement of the films while also creating microdefects or pores due to their uneven dispersion [44]. This dispersion could help improve the multifunctionality of the films, such as UV resistance and antibacterial properties.

### 3.7. The Mechanical Properties of Films

**Figure 9:**
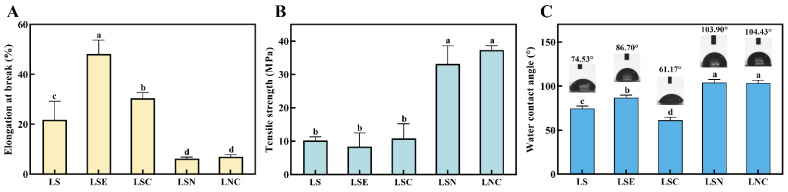
The TS (**A**), EB (**B**), and WCA (**C**) of different films. Different lowercase letters in the same column indicate significant differences (*p* < 0.05).

The tensile strength (TS) and elongation at break (EB) of the films are used to assess their mechanical properties. The EB and TS of films with different compositions are shown in Figure 9A,B. In terms of EB values, the LSE film with honeysuckle essential oil had the highest EB value, reaching 48.19 ± 4.80%. This may have been due to the essential oil weakening the cohesion between the large molecules of lily starch [45]. The inclusion of the oil phase influenced the film’s continuous and stable 3D structure, facilitating the displacement of starch polymer chains. Acting as a plasticizer, it enhanced the film’s toughness and resulted in a higher elongation at break (EB) value. In contrast, the LSN and LNC films with cellulose nanofibers showed poor toughness, with EB values of 6.35 ± 0.44% and 6.96 ± 0.77%, respectively. This was because the added CNF are crystalline fibers with high rigidity, which increased the strength of the film but reduced its toughness [46]. Similar findings have been reported in previous studies, where the incorporation of CNF led to a decrease in elongation at break due to its rigid nature [47,48]. For TS, the films with CNF, LSN and LNC, achieved the highest TS values, 33.15 ± 2.78 MPa and 37.31 ± 0.79 MPa, respectively, while the pure lily starch film, LS, had only 10.17 ± 1.04 MPa. The increases were 226.6% and 267.3%, respectively, which proved that the rigid effect of CNF significantly enhanced the mechanical properties of the films. Yi-Ting Shih’s study has also demonstrated that CNF can serve as an effective reinforcement, significantly improving the tensile strength of biopolymer films [49].

### 3.8. Water Contact Angle of Films

The water contact angle (WCA) is a widely used method to assess the hydrophilic or hydrophobic nature of the film surface. A high WCA value (θ ranging from 90° to 180°) indicates that the film exhibits hydrophobic properties [50]. In food packaging, materials with high hydrophobicity could act as a water barrier, preventing contamination of the internal food products. As shown in Figure 9C, the pure LS film, due to the hydrophilic nature of starch, had a WCA of 74.53 ± 2.30°, which showed it was relatively hydrophilic [51]. After adding honeysuckle essential oil, the WCA of the LES film increased slightly to 86.7 ± 2.50° because of the oil’s hydrophobicity, but it still did not reach the hydrophobic surface standard. On the other hand, after adding CZH hybrid particles to LS, the WCA decreased significantly to 61.17 ± 2.88°. Chitosan, with many hydroxyl groups, formed hydrogen bonds with water molecules, which made the water contact angle smaller. However, the films with CNF, LSN and LNC, showed good hydrophobicity, with WCAs of 103.9 ± 2.92° and 103.43 ± 2.64°, respectively. This result was similar to previous studies and showed that adding CNF improved the hydrophobicity of the films and reduced water penetration [52].

### 3.9. Barrier Properties of Films

**Figure 10:**
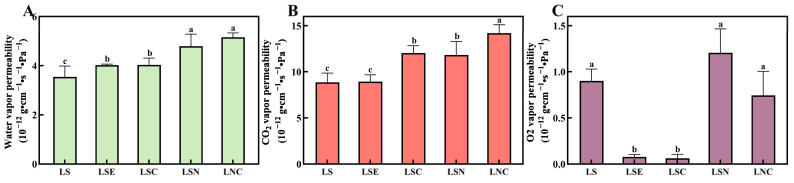
Water vapor permeability (**A**), CO_2_ vapor permeability (**B**), and O_2_ vapor permeability (**C**) of different films. Different lowercase letters in the same column indicate significant differences (*p* < 0.05).

Barrier properties, such as H_2_O vapor permeability, CO_2_ vapor permeability, and O_2_ vapor permeability, offer crucial insights into the potential application of films in food packaging. These properties play a significant role in determining the film’s ability to protect the food from external environmental factors and help preserve its quality [53]. These properties are needed differently for different foods. For example, for fruits and vegetables, higher permeability is required to allow gas exchange, control respiration, and prevent water vapor from condensing. For foods that are easy to oxidize or absorb moisture, like dried foods or jerky, it is important to strictly control the entry of water vapor and oxygen [54]. The barrier properties of the films are shown in Figure 10.

The pure lily starch film LS has low water vapor permeability. After adding different materials, the water vapor permeability of the films changed. The film LSE, with honeysuckle essential oil added, slightly improved the water vapor barrier performance [55]. This was likely because the hydrophobicity of the essential oil increased the film’s resistance to water. However, after adding the hybrid particles CZH, the water vapor permeability of the LSC film increased further. This was possibly because the hydrophilic groups in chitosan interacted more with water molecules, reducing the barrier effect. In contrast, the films LSN and LNC with CNF showed better water vapor permeability. Although CNF had high crystallinity and hydrophobicity, which made the surface more water resistant, its combination with CZH may have changed the microstructure of the film, creating more permeation channels and increasing water vapor permeability [56].

The CO_2_ barrier performance showed clear differences between the films. The LS film had the lowest CO_2_ permeability, showing that pure lily starch had good CO_2_ barrier properties. After adding CZH hybrid particles, the CO_2_ permeability of the film increased significantly [57]. This was possibly because the intermolecular interactions of chitosan made the film structure looser, creating more gas diffusion channels. The films with CNF (LSN and LNC) showed higher CO_2_ permeability, with LNC reaching the highest value of 14.19 ± 0.81. This showed that, although CNF improved the mechanical properties of the film, it had a negative effect on the CO_2_ barrier performance.

For O_2_ barrier performance, the LS film showed high O_2_ permeability, meaning pure lily starch had weak oxygen resistance. Adding honeysuckle essential oil and CZH hybrid particles significantly reduced the O_2_ permeability of the LSC and LNC films. The films with CNF (LSN and LNC) had the best oxygen barrier performance. This was likely because CNF had high crystallinity and a tight network structure, which effectively blocked O_2_ molecules from passing through.

## 4. Conclusions

Lily starch, as a natural polysaccharide, is abundant in resources, but its application in packaging materials has been rarely studied. Therefore, this study used lily starch as the base material to develop a new biodegradable functional packaging film. The film was systematically optimized, showing excellent film-forming properties and good compatibility with other functional components. GC-MS analysis identified the main components of honeysuckle essential oil. The essential oil was effectively integrated into the film as chitosan–ZnO@honeysuckle essential oil hybrid particles. Cellulose nanofibers were incorporated to enhance the mechanical properties and ensure the structural uniformity of the film. This study demonstrated the potential of lily starch and honeysuckle essential oil in green food packaging and provided a theoretical basis for developing green, high-performance packaging materials. In future research, we will focus on optimizing the scaling-up process and exploring the practical industrial applications of these films, including investigating the long-term stability and performance of the developed packaging materials under real-world conditions. In addition, we will ensure that the environmental impact and regulatory compliance of the proposed films are thoroughly addressed, in line with current sustainability standards and regulatory frameworks.

## Figures and Tables

**Table 1:** The Optical performance parameters L, a, b, and ΔE of different films. Different lowercase letters in the same column indicate significant differences (*p* < 0.05). LS—Pure lily starch film; LSE—Lily starch film incorporated with honeysuckle essential oil; LSC—Lily starch film containing chitosan–ZnO–honeysuckle essential oil hybrid particles (CZH); LSN—Lily starch film incorporated with cellulose nanofibers (CNF); LNC—Lily starch film containing CNF and CZH.

Film	L	a	b	∆E
LS	35.51 ± 0.56 ^b^	−0.15 ± 0.05 ^a^	−1.55 ± 0.20 ^a^	58.05 ± 0.57 ^a^
LSE	35.55 ± 1.81 ^b^	−0.12 ± 0.06 ^a^	−1.51 ± 0.37 ^a^	58.91 ± 1.80 ^a^
LSC	40.11 ± 1.73 ^a^	−0.31 ± 0.15 ^b^	−6.04 ± 0.85 ^b^	53.65 ± 1.60 ^b^
LSN	35.60 ± 0.36 ^b^	−0.07 ± 0.05 ^a^	−1.01 ± 0.29 ^a^	58.04 ± 0.35 ^a^
LNC	40.88 ± 2.24 ^a^	−0.50 ± 0.18 ^c^	−5.98 ± 0.72 ^b^	53.62 ± 2.13 ^b^

## Data Availability

The original contributions presented in the study are included in the article, further inquiries can be directed to the corresponding author.
